# A study of an intelligent system to support decision making in the management of labour using the cardiotocograph – the INFANT study protocol

**DOI:** 10.1186/s12884-015-0780-0

**Published:** 2016-01-20

**Authors:** Peter Brocklehurst, on behalf of The INFANT Collaborative Group

**Affiliations:** UCL EGA Institute for Women’s Health, 74 Huntley Street, WC1E 6 AU London, UK

**Keywords:** Monitoring, Labour, Computerised, Interpretation, Decision support, Cardiotocogram, Continuous electronic fetal monitoring

## Abstract

**Background:**

Continuous electronic fetal heart rate monitoring in labour is widely used but its potential for improving fetal and neonatal outcomes has not been realised. The most likely reason is the difficulty of interpreting the fetal heart rate trace correctly during labour. Computerised interpretation of the fetal heart rate and intelligent decision-support has the potential to deliver this improvement in care.

This trial will test whether the addition of decision support software to aid the interpretation of the cardiotocogram (CTG) during labour will reduce the number of ‘poor neonatal outcomes’ in those women judged to require continuous electronic fetal heart rate monitoring.

**Methods and design:**

An individually randomised controlled trial of 46,000 women who are judged to require continuous electronic fetal monitoring in labour.

*Eligibility criteria:* Women admitted to a participating labour ward who are judged to require continuous electronic fetal monitoring, have a singleton or twin pregnancy, are ≥ 35 weeks’ gestation, have no known gross fetal abnormality and are ≥ 16 years of age.

*Exclusion criteria:* Triplets or higher order pregnancy, elective caesarean section prior to the onset of labour, planned admission to NICU.

*Trial interventions:* Computerised interpretation of the CTG with decision-support.

Primary outcomes: *Short term:* A composite of ‘poor neonatal outcome’ including stillbirth after trial entry, early neonatal death except deaths due to congenital anomalies, significant morbidity: neonatal encephalopathy, admissions to the neonatal unit with 48 h for > 48 h with evidence of feeding difficulties, respiratory illness or encephalopathy where there is evidence of compromise at birth. *Long term:* Developmental assessment at the age of 2 years in a subset of 7000 surviving babies.

*Data Collection:* For all participating women and babies, labour variables and outcomes will be stored automatically and contemporaneously onto the Guardian® system.

**Discussion:**

The results of this trial will have importance for pregnant women and for health professionals who provide care for them.

**Trial registration:**

Current Controlled Trials ISRCTN98680152 assigned 30.09.2008

## Background

Continuous electronic fetal heart rate monitoring (EFM) in labour is widely used throughout the developed world. However, its potential for improving fetal and neonatal outcomes has not been realised. The reasons for this are complex, but the most likely reason is the difficulty of interpreting the fetal heart rate trace correctly during labour, when the birth attendant has many competing tasks. If intrapartum monitoring is ever going to improve fetal and neonatal outcomes then the interpretation of the fetal heart rate has to be substantially improved. This improvement then has to be sustained and be independent of any health professional’s individual ability. Computerised interpretation of the fetal heart rate and intelligent decision-support has the potential to deliver this improvement in care. The aim of EFM is to detect abnormalities of the fetal heart rate pattern during labour that are associated with asphyxia so that action can be taken to expedite delivery and prevent stillbirth and the development of encephalopathy. Therefore the potential benefits of EFM are immense. Prevention of even a modest proportion of perinatal asphyxia will improve the health and well-being of thousands of children and their families throughout the world each year. In addition the NHS Litigation Authority bill for obstetrics was £269 m in 2001/2 and is rising. This could be substantially reduced. Furthermore, if this technology can work in the complex process of labour it also has the potential to improve patient safety in a wide range of health-care settings.

### The problem of perinatal asphyxia

Perinatal asphyxia, if severe, can result in intrapartum stillbirth. If less severe it results in the development of an encephalopathic state in the newborn. This is characterised by a decreased level of consciousness, altered reflexes and abnormal tone and ultimately permanent damage to the brain. Moderate or severe neonatal encephalopathy occurs in approximately 2/1000 births [1]. With more severe asphyxial encephalopathy there is an increasing risk of death or neurodevelopmental abnormalities: up to 30 % of affected neonates will develop seizures and approximately 25 % of infants who have moderate asphyxial encephalopathy will develop cerebral palsy. Almost all infants with severe encephalopathy die or survive with multiple handicaps [2]. Perinatal asphyxia may account for up to 30 % of cases of cerebral palsy [3] and it is a very significant health-care and financial burden on the NHS. A reduction in the number of babies born with perinatal asphyxia would reduce the associated mortality and, amongst survivors, the burden of ill health and incapacity. It could also result in substantial savings in litigation costs in the UK.

### Efficacy of continuous electronic fetal monitoring (EFM)

Continuous EFM was invented in the 1960s [4, 5]. The recorder displays the fetal heart rate and maternal uterine activity and displays this on a continuous line graph, called the cardiotocograph (CTG) tracing. EFM was widely introduced in the 1970s [6]. It became controversial in the 1980s when it was shown that it was poorly predictive of Apgar scores and fetal acid–base status at delivery [7]. The largest randomised controlled trial (the Dublin trial) showed no reduction in perinatal mortality or in cerebral palsy using EFM [8]. However, systematic reviews and meta-analyses of all trials indicated some benefits of continuous EFM: for example, a 58 % reduction in odds of deaths attributable to intrapartum hypoxia (95 % confidence interval, 2 to 83 %) [9], (see Table 1), and a 50 % reduction in risk of neonatal seizures (95 % confidence interval, 20 to 69 %) [10]. EFM is widely used on many women during labour in the UK. National Institute for Health and Care Excellence (NICE) guidelines for fetal monitoring in the NHS detail explicit criteria for which women should have continuous EFM during labour; these equate to approximately 60 % of all women in labour [11].

**Table 1:** The effect of continuous EFM versus intermittent auscultation on the incidence of deaths attributable to intrapartum hypoxia

	No. of patients in the EFM group	No. of patients in the IA group	No. of perinatal deaths		Perinatal deaths due to fetal hypoxia	
Study			EFM	IA	EFM	IA
Haverkamp *et al* (1976) [45]	242	241	2 (FD 0, ND 2)	1 (FD 0, ND 1)	0	0
Renou *et al* (1976) [46]	175	175	1 (FD 0, ND1)	1 (FD 1, ND 0)	0	1 (FD)
Kelso *et al* (1978) [47]	253	251	0	1 (FD 0, ND 1)	0	1 (ND)
Haverkamp *et al* (1979) [48]	230 229	231	3 (FD 0, ND 3)	0	0	0
Wood *et al* (1981) [49]	445	482	1 (FD 0, ND 1)	0	0	0
MacDonald *et al* (1985) [8]	6474	6490	14 (FD 3, ND 11)	14 (FD 2, ND 12)	7 (FD 3, ND 4)	7 (FD 2, ND 5)
Neldam *at al* (1986) [50]	482	487	0	1 (FD 1, ND 0)	0	1 (FD)
Luthy *et al* (1987) [51]	122	124	17 (FD 1, ND 16)	18 (FD 1, ND 17)	0	1 (FD)
Vintzileos *et al* (1993) [52]	746	682	2 (FD 0, ND 2)	9 (FD 2,ND 7)	0	6 (FD 2, ND 4)
Total	9398	9163	40 (4.2/1000)	45 (4.9/1000)	7 (0.7/1000)^a^	17 (1.8/1000)^a^

*EFM* electronic fetal monitoring, *IA* intermittent auscultation, *FD* fetal (intrapartum) death, *ND* neonatal death

^a^Statistically significant difference; Mantel-Haenszel odds ratio 0.42 (95 % confidence interval 0.17 to 0.98)

### Human error and systems failure

In the late 1980s it became apparent that a human element might be a factor in EFM failing to deliver an improved outcome. In one case–control study, the intrapartum management of 38 babies severely asphyxiated at birth was compared with that of 120 controls [12]. In the control group, 29 % of babies had an abnormal CTG, but in only 9 % was the abnormality severe. In contrast, 87 % of the babies asphyxiated at birth had an abnormal CTG and in 61 % of cases the abnormality was severe. The most striking finding, however, was the length of time required for the staff to recognise the CTG abnormality. With moderate abnormalities, the mean time to recognition was 91 min (SD 61); paradoxically, with severe abnormalities it was 128 min (SD 100). The authors could give no plausible reason for the standard of CTG interpretation being so poor. However, it was clear from this study that if the quality of interpretation of the intrapartum CTG had been higher, the benefits from EFM would almost certainly have been significantly and substantially enhanced.

In 1990, Ennis and Vincent published the results of their study of 64 cases of poor perinatal outcome from the archives of the Medical Protection Society [13]. In 11 cases continuous EFM was not performed, although indicated; in six cases the technical quality of the tracing was inadequate; in 14 cases there was a significant abnormality in the CTG, but this was either not noticed or no action was taken upon it; in only 14 cases was appropriate monitoring performed and action taken; and the CTG was missing in 19 cases. In only 16 cases was a consultant involved to aid in the interpretation of the CTG. In a further study from Oxford published in 1994, intrapartum care was assessed in 141 cases of cerebral palsy and in 62 perinatal deaths with a likely intrapartum cause [14]. The authors found that abnormal fetal heart-rate patterns were: 2.3 times as common in babies who went on to develop cerebral palsy, compared with controls; and 6.7 times as common in perinatal deaths. In addition, the authors found that clinicians’ failure to respond to these clear signs of abnormality occurred in 26 % of cerebral palsy cases and 50 % of perinatal deaths, compared with 7 % of controls. On the basis of these figures it can be estimated that approximately one case of cerebral palsy and one perinatal death can possibly be prevented in every 4500 deliveries. If one assumes 700,000 births per annum in the United Kingdom, 174 cases of cerebral palsy and 158 perinatal deaths could be prevented each year. More recently, Stewart et al have reported that perinatal mortality in the United Kingdom is twice as high at night as during the day, and twice as high in July and August as in the rest of the year [15]. They suggest that excess deaths may be due to over-reliance on inexperienced staff at night and a shortage of staff during the peak summer holiday months; they also suggest that the excess might be related to physical and mental fatigue of the caregivers. In 1999 the Confidential Enquiry into Stillbirths and Deaths in Infancy (CESDI) studied the proportion of 567 cases where there was evidence of suboptimal care in labour. CESDI then looked at whether improved care could possibly or probably have prevented the adverse outcome [16]. Suboptimal care was identified in 71 % of cases; a better outcome could possibly (in 28 % of cases) or probably (in 22 % of cases) have been anticipated, if care had been adequate. The report commented that “fetal surveillance problems were the most common cause [of problems in labour], with CTG interpretation the most frequent criticism.”

### Does improving training solve the problem?

In a report of a study of the efficacy of intrapartum intervention, Young et al found that when babies with low Apgar scores were studied, in 74 % there was evidence of substandard care in labour [17]. Following the introduction of regular audit of low Apgar scores, with intensive feedback to clinical staff, this proportion fell to 23 %, but increased to 32 % over the following year. However, following the introduction of compulsory training in CTG interpretation for all staff, the proportion of low Apgar score cases associated with substandard care fell back once again to only 9 %. It is clear from this study that improved interpretation of CTGs during labour can bring about a striking increase in the quality of care, with measurable impacts on neonatal condition. However, intensive education is not sustainable in most clinical settings. With recent changes in the training of junior medical and midwifery staff, it is clear that other systems have to be developed which are less reliant on individual motivation and training. These systems need to work equally well, regardless of the time of day, day of the week, month of the year, and the level of staffing on the labour ward.

### Litigation and the costs to families and society

Maternity services are associated with far higher litigation costs than other services (http://news.bbc.co.uk/1/hi/england/beds/bucks/herts/6310805.stm). This is reflected in the various arrangements for the development of risk management standards across the UK (Clinical Negligence Scheme for Trusts in England, Welsh Risk Pool, Clinical Negligence and Other Risks Indemnity Schemes and NHS Quality Improvement Scotland in Scotland) [18].

Payments made (including amounts set aside for unresolved claims) by the National Health Service Litigation Authority for obstetric related incidents over the period 1995 to 2005 totalled £1.5 billion.

In response to a parliamentary question on 29 Jan 2007, it was stated that the total compensation payout in 2006 was £593 million, with £68 million resulting from just ten cases, all of which were in relation to pregnancy and childbirth. The BBC also reported a settlement of £6 million for a child with cerebral palsy after doctors “mis-managed her delivery” (http://news.bbc.co.uk/1/hi/england/beds/bucks/herts/6310805.stm). Even successful defence can cost up to £0.5million. Not surprisingly, the Chief Medical Officer set a target19 of a 25 % reduction in obstetric mishaps by the year 2005. However in his 2006 report there was still a chapter devoted to intrapartum related deaths [19]. The BMJ in 2000 highlighted the importance of “system errors” in medical disasters [20]; analogies were drawn with errors in aviation. It has been suggested that some techniques used in this industry could be applied effectively to medical care, such as safety drills, revalidation, ‘near miss’ reporting and a ‘no blame’ culture. The role of expert systems and ‘intelligent alarms’ was highlighted.

### The potential solution: development of the intelligent decision-support software

A group in Plymouth, working on the problems of resolving human error in the management of labour, have developed intelligent computer systems as decision aids to support clinicians. The group were funded by the MRC (G9721800) for development and clinical validation of a decision-support tool for the management of labour using the CTG. This decision support software interprets the CTG in the context of an individual woman’s labour and offers advice on the management of labour. It comprises feature extraction of all relevant data from the CTG and clinical history which have been found to influence clinicians’ decision making, and then an analysis of these within a rule-based expert system.

The specific piece of decision-support software to be evaluated in INFANT has been designed by K2 Medical Systems (a spin-off company from the University of Plymouth) to run on the K2 data collection system (Guardian®). The data collection system (Guardian®) is a system for managing information from labour monitoring. It displays the CTG on a computer screen alongside other clinical data which are collected as part of routine clinical care. As such, it replaces conventional paper labour notes, the CTG machine and other recording systems for documenting care during labour. The data collection system (Guardian®) does not interpret any of the data being collected, it merely acts as an interface to collect and display data. If used to its full potential it results in the labour room being a notes-free area.

#### The data collection system (Guardian)

The Guardian system consists of a medical-grade PC platform, which meets the MHRA standards for a class IIa device. The design has been informed by user-preference studies and ethnographic and audio-visual observations of clinical care and decision making [21, 22]. It has a touch-screen user-interface www.K2ms.com and is connected to a conventional CTG recorder at the woman’s bedside.

**Fig. 1:**
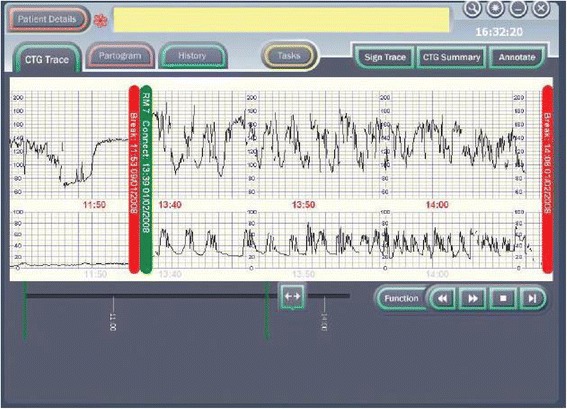
The data collection system (Guardian) displays the CTG data on the screen

The PC uses the Windows operating system and runs the decision-support software developed by the Plymouth Group. The clinician enters clinical information (antenatal risk factors, vaginal examination data, fetal blood sample results, etc.) via the touch-screen. This information is displayed as a partogram. The system requires little or no training to use and has been used for routine clinical care by a number of hospitals throughout the UK [23]. If a CTG is performed by ultrasound or ECG clip, the PC system automatically collects this from the RS 232 digital data-port of any CTG recorder. The system displays the CTG data on the screen (Fig. 1).

#### The decision-support software

The decision-support software is a specific piece of software which has been developed to run on the Guardian® system. It extracts the important features of baseline heart rate, heart-rate variability, accelerations, type and timing of decelerations, the quality of the signal and the contraction pattern from the CTG. The decision-support software then analyses these data along with the quality of the signals. The system’s assessment of the CTG is presented as a series of colour-coded alerts depending on the severity of the abnormality detected. The system can therefore be viewed as an intelligent prompt, but by recording the chronology of events it also offers the opportunity to later audit the actual clinical decision-making process in a similar way to an airliner’s black box.

### Studies using the intelligent support software

Three studies conducted by the Plymouth group [24–26] have demonstrated that the software, when used ‘off-line’, performed as well as expert obstetricians in interpreting the CTG and managing labour subsequently, and that the system performed better than routine clinical practice. The system identified more cases that went on to have a poor outcome and anticipated clinical decision-making. In one of these studies involving labours that had resulted in a stillbirth, the system “intervened” more than six hours earlier than actual clinical practice, and more than two hours before the experts. If this translated into clinical practice, it would be reasonable to expect that a number of such deaths might have been prevented if the software had been in use at the time. In all other poor-outcome groups the system intervened much earlier than had happened in routine clinical practice and at a similar time as the experts. The system failed to predict one perinatal death, whereas the experts in the ‘off line’ study and those functioning in routine clinical practice failed to predict several deaths. These extensive ‘off-line’ validation studies have shown that the system matched the performance of an expert obstetrician in interpreting the CTG, and performed considerably better than routine clinical practice. Further, the system is not over-interventional. From these data it seems reasonable to hypothesise that the clinical use of this computer-based decision-support software will decrease the incidence of perinatal mortality and morbidity.

#### Current practice

EFM is widely used for the majority of women during labour and birth in the UK. NICE guidelines for fetal monitoring detail explicit criteria indicating which women should be offered continuous EFM during labour; approximately 60 % of all women in labour meet these criteria [11]. This study will not influence the number of women who receive continuous EFM.

#### Research objectives

The objectives of the study are:

1. to determine whether intelligent decision-support can improve interpretation of the intrapartum cardiotocograph (CTG) and therefore improve the management of labour for women who are judged to require continuous electronic heart rate monitoring. Specifically, will the system, compared with current clinical practice:
  i. identify more clinically significant heart rate abnormalities?
  ii. result in more prompt and timely action on clinically significant heart rate abnormalities?
  iii. result in fewer “poor neonatal outcomes”?
  iv. change the incidence of operative interventions?
2. to assess whether use of intelligent decision-support improves the quality of routine care received by women undergoing continuous electronic fetal monitoring during labour. This information will be important for evaluating whether the decision-support software decreases the risk of suboptimal care in labour; it will also be useful to explore the effect that such an intervention may have on litigation for obstetrics.
3. to determine whether the use of the decision-support software is cost-effective in terms of the incremental cost per poor perinatal outcome prevented.
4. to determine whether use of the decision-support software has any effect on the longer term neurodevelopment of children born to women participating in the INFANT study.

## Methods/Design

### Research methods

An individually randomised controlled trial of 46,000 women who are judged to require continuous electronic fetal monitoring in labour.

Follow-up at age 2 years of a sample of 7000 surviving children born to women participating in the INFANT study.

### Trial eligibility and randomization

Women admitted to a participating labour ward who fulfil all of the following criteria will be eligible to be randomised if:

- they are judged to require continuous electronic fetal monitoring (EFM) by the local clinical team based on their existing guidelines, and the woman consents to have EFM, and EFM is possible
  *Note: continuous EFM is defined as the active decision of the health care professional and the woman to initiate continuous EFM for the purpose of fetal monitoring, usually because of a perceived risk factor(s) which increases the likelihood of fetal compromise occurring in labour. The Guardian system will prompt the health care professional to consider women eligible for the INFANT trial if the CTG is used.*
  *The decision to initiate continuous EFM can occur at any time during labour. Some women with known factors which place them at higher risk of fetal compromise during labour will already know that continuous fetal monitoring throughout labour is planned. Others will start off labour with intermittent monitoring and then be judged to require continuous fetal monitoring at some point during the labour. There is no point during labour at which women are not eligible to participate (this includes during the second stage).*
- they have a singleton or twin pregnancy
- they are ≥ 35 weeks’ gestation (≥245 days)
- there is no known gross fetal abnormality, including any known fetal heart arrhythmia such as heart block
- they are 16 years of age or older
- they are able to give consent to participate in the trial as judged by the attending clinicians.

#### Randomisation

The Guardian® system will prompt the health professional providing care to consider whether the woman is eligible for the INFANT trial, when EFM has been used for more than 5 min. Intermittent use of EFM for periods of up to 5 min duration may be used for intermittent monitoring, but when used for longer periods of time this will often indicate that a decision has been made to initiate continuous EFM, in which case the women may be eligible to participate in the trial. If the health care professional indicates that the women is not yet eligible because an active decision has not been made to initiate continuous EFM, then the Guardian® system will prompt the health care professional again, if the CTG continues to be recorded for longer than 5 min in this or any subsequent episode of monitoring.

When the health care professional indicates that a woman is eligible to participate, the Guardian® system will clarify that the necessary eligibility criteria for trial entry have been met, i.e. that the health professional gives the required answers to a number of questions posed by the Guardian® system and then the Guardian® system will randomly allocate the women in the ratio 1:1 to either “CTG with no decision-support” or “CTG with decision-support”. The allocations will be computer generated in Stata software (release 10) using stratified block randomisation employing variable block sizes to balance between the two trial arms by whether the pregnancy is a singleton or twins, and within each participating centre. The procedures for randomisation will be fully documented, reviewed and signed off prior to the start of the trial and monitored by the co-ordinating centre during the trial.

As all de-identified information collected by the Guardian® system within the participating centre can be accessed centrally, the trial co-ordinating centre and the participating centres will be able to monitor the performance of centres in terms of the number of women the Guardian system considers eligible who are recruited to the trial to ensure that the necessary systems within the centre are optimised to maximise recruitment.

### Exclusion criteria

- triplets or higher order pregnancy
- criteria for EFM not met, including elective caesarean section prior to the onset of labour

### Planned interventions

The intervention is the decision-support software in this trial. In order to accurately reflect any potential impact of the decision-support software in contemporary NHS practice, such as changes in midwifery presence during labour consequent upon knowledge of the allocation, it is desirable that clinicians are not masked to allocation.

### Clinical management

The Guardian system will usually be used by all women in labour in the participating centres. It is only the decision-support software, which runs on this system, that is being tested in this trial.

**Fig. 2:**
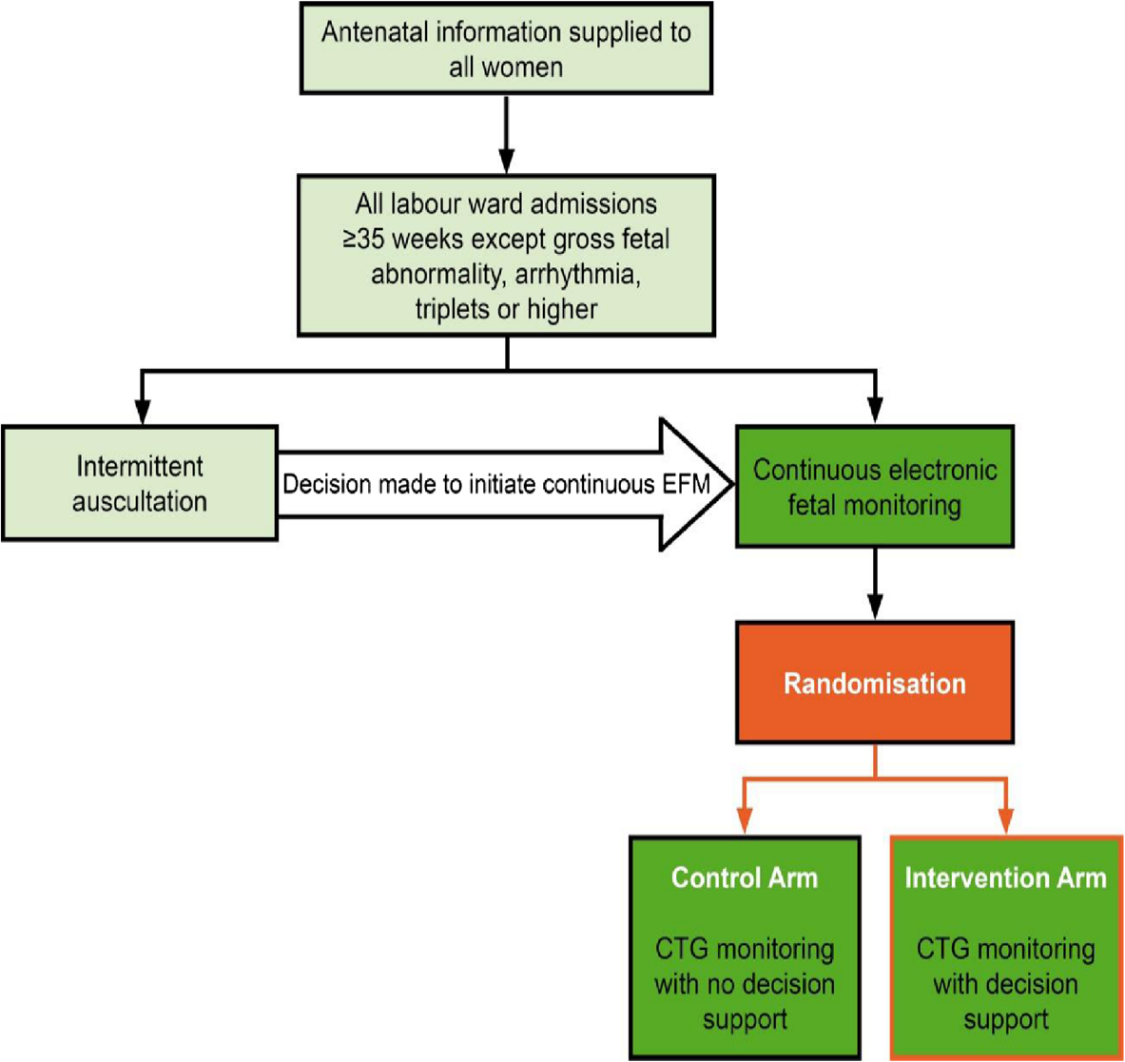
Clinical Management flow diagram

The clinical management of women participating in this trial will remain unaltered by their participation, apart from the relatively uncommon circumstances when abnormalities of the CTG prompt the system in the ‘decision-support’ arm of the trial to issue a series of alerts or alarms, which will increase in urgency with the severity of the abnormality (See Fig. 2).

Information about the trial will be provided to all women during the antenatal period, after their booking appointment. This process will be individualised for each participating centre depending on their routine practices. For example, in some centres, women will be provided with information about the trial at their routine ultrasound scan appointment (18–22 weeks). All women will have the opportunity to ask questions.

When a woman presents in early labour to the labour ward in a participating centre, she will be given a copy of the Participant Information Leaflet and a verbal explanation of the INFANT trial. She will then be asked whether she would like to participate in the study and if she agrees she would be asked to sign an INFANT trial consent form. If then at any point continuous electronic fetal monitoring (EFM) is commenced, then the midwife responsible for her care will check her eligibility to participate in the trial and check that the woman is still happy to take part and document this, then the woman will be randomised by the Guardian® system to either decision-support (intervention arm) or no decision-support (control arm).

All women admitted in labour to the participating centres will usually have their labour information recorded in the Guardian® system, according to the current practice in each centre. This does not change the way health professionals manage labour, it merely changes the way they manage the information generated by the process of monitoring labour and how they record this information. It will be clearly stated that women are free to withdraw from the study at any time for any reason without prejudice to future care, and with no obligation to give the reason for withdrawal.

Written informed consent will be obtained by means of a dated signature from the woman and the signature of the person who obtained informed consent; this would be the Principal Investigator (or a qualified health care professional with delegated authority). A copy of the signed informed consent document will be given to the women. A further copy will be retained in the woman’s medical notes, a copy will be retained by the Principal Investigator and a final copy will be sent to the Trial Coordinating Centre.

A senior investigator will be available at all times to discuss concerns raised by women or clinicians during the course of the trial.

### Outcome measures

The primary outcomes of this trial will be:

#### Short term

A composite of ‘poor neonatal outcome’ to include (a) all deaths *(intrapartum stillbirths plus neonatal deaths* i.e. *deaths up to 28 days after birth)* except deaths due to congenital anomalies, (b) significant morbidity: neonatal encephalopathy (moderate and severe); (c) admissions to the neonatal unit within 48 h of birth for ≥ 48 h with evidence of feeding difficulties, respiratory illness or encephalopathy where there is evidence of compromise at birth. (We recognise that the signs of mild encephalopathy can be subtle and hence a number of such babies are likely to have a range of non-specific signs such as respiratory difficulty and poor feeding rather than features more specifically associated with encephalopathy [27]. Hence including admission to the neonatal unit within 48 h of birth for equal to or greater than 48 h where there is evidence of compromise at birth. Since this is a mature group of babies, any difference in the incidence of these admissions is likely to result from differences in perinatal asphyxia).

*Note: the benefit of using a composite outcome is the increased incidence of the outcome; hence the sample size is reduced. The main problem occurs if the intervention affects different outcomes in different ways. In this trial, if the use of the decision-support software decreased deaths but increased significant morbidity there may be no difference in the short term primary outcome between the two arms of the trial, even though deaths were being prevented in one arm. The probability of this situation occurring appears very small. If decision-support prevents perinatal asphyxia it will have an effect on all the outcomes contained within the combined primary outcome.*

#### Long term

PARCA-R composite score [28, 29] at the age of 2 years for all infants with trial primary outcome and a further subset of 7000 babies.

*Note: Neurodevelopmental delay and cerebral palsy are the most important long-term adverse outcomes associated with perinatal asphyxia. However the incidence of moderate or severe cerebral palsy is of the order of 1.5–2.5/1000 live births, depending on the definition and the method of ascertainment. There is also uncertainty about the proportion of these cases that results from intrapartum asphyxia in mature infants; however, 30 % is a reasonable estimate3. Therefore, given the rarity of this outcome, it is unlikely that a clear difference could be demonstrated between the two groups with a trial of 46,000 births. So in order to have reassurance that any benefits of the intervention, with respect to short term outcomes, have not occurred at the expense of later neurodevelopmental delay,we will measure neurodevelopment in a proportion of the surviving children at the age of 2 years.*

#### Secondary outcomes

- Intrapartum stillbirth except deaths due to congenital anomalies
- Neonatal deaths up to 28 days after birth except deaths due to congenital anomalies
- Moderate or severe encephalopathy
- Admission to neonatal unit within 48 h of birth for ≥ 48 h with evidence of feeding difficulties, respiratory illness or encephalopathy (where there is evidence of compromise at birth)
- Admission to a higher level of care
- Apgar score <4 at 5 min
- The distribution of cord blood gas data for cord artery pH
- Metabolic acidosis (defined as cord artery pH <7.05 and base deficit in extracellular fluid ≥ 12 mmol/l)
- Resuscitation interventions
- Seizures
- Destination immediately after birth
- Length of hospital stay

Health and development outcomes at 24 months (for all infants with trial primary outcome and a further subset of 7000 babies):

- ▪ Non-verbal Cognition Scale (PARCA-R)
- ▪ Vocabulary Sub-scale (PARCA-R)
- ▪ Vocabulary Sub-scale (PARCA-R)
- ▪ Sentence Complexity Sub-scale (PARCA-R)
- ▪ Late deaths up to 24 months (after the neonatal period)
- ▪ Major disability and non-major disability at 2 years
- ▪ Cerebral palsy
- ▪ Breast feeding (collected at 12 and 24 months)

Maternal

- Mode of delivery
- Operative intervention (caesarean section and instrumental delivery) for (i) fetal indication, or (ii) failure to progress, or (iii) combination of fetal distress and failure to progress, or (iv) other reason.
- Grade of Caesarean section
- Episiotomy
- Any episode of fetal blood sampling
- Length of (i) first stage, (ii) second stage and (iii) total length of labour from trial entry
- Destination immediately after birth
- Admission to a higher level of care

#### Quality of care

All babies with an adverse outcome (trial primary outcome plus cord-artery pH <7.05 with base deficit ≥ 12 mmol/l) and all neonatal deaths and intrapartum stillbirths will have their care in labour assessed to see if it was suboptimal. All cases of adverse neonatal outcome (primary outcome plus cord-artery pH <7.05 with base deficit ≥ 12 mmol/l) will undergo panel review similar to that undertaken by CEMACH [16]. Intrapartum notes will be copied and anonymised, and all references to trial allocation will remain. These notes will then be examined by a panel of experienced obstetricians, midwives and neonatologists to identify if there was suboptimal care, particularly in relation to interpretation of the CTG and actions which flow from any identification of CTG abnormalities. The panels will also seek to identify cases with clear evidence of suboptimal care, where the care could be considered negligent in the event of litigation. This information will be important for evaluating whether the decision-support software decreases the risk of suboptimal care in labour; it will also be useful in the economic modelling, which will seek to explore the effect that such an intervention may have on litigation in obstetrics.

#### Process outcomes

It is important to collect and analyse process outcomes in this trial, as a failure to detect differences in clinical or quality of care outcomes between the two randomised groups may be due to poor compliance with the alerts of the system, rather than that the system did not correctly identify abnormalities with the CTG. In addition, as the trial allocation is not masked, it will be important to measure any change that results from clinicians being aware of whether the decision-support system is in operation or not. The following outcomes will therefore be measured to assess this:

- Proportion of women with a CTG abnormality (as identified by the study software)
- Number of CTG abnormalities identified in the two arms (as identified by the study software)
- Time taken between alerts and delivery. This can be achieved in the ‘no decision-support’ arm by using the decision-support software to analyse the CTG trace after the trial is over and using this to determine when the alert would have occurred.
- Number of routine measurements recorded during labour, including the number of vaginal examinations, use of epidural analgesia, use of labour augmentation and presence of meconium.
- Number of thumb entries per hour from time of trial entry to first yellow level of concern or until fully dilated
  *These are proxy measures to assess presence of a health professional in the delivery room during the labour which will allow us to quantify any important differences between the groups with respect to support offered to women during labour. Although unlikely, knowledge of the trial allocation may result in less frequent contact with the woman allocated ‘decision-support’ in labour. Less frequent contact will result in a lower number of these process measures.*

#### Data collection

For all participating women and babies, labour variables and outcomes will be stored automatically and contemporaneously onto the Guardian® system. Data collected via the system will be sent electronically to the trial co-ordinating centre at the UCL Clincial Trials Unit (UCL CTU) in London. Data will be extracted from the notes of babies admitted to the neonatal unit and for all neonatal deaths. It should be noted that not all data fields are collected at every centre. However, where an item is collected these data will be sent to the UCL CTU. The trial is not collecting the reason why continuous EFM is being used, as this is not recorded. All children surviving to be discharged home from hospital following their birth will be ‘flagged’ at the NHS Information Centre for those born in England and Scotland and all deaths occurring after discharge home from hospital will be notified to the trial co-ordinating centre at the UCL CTU. At 2 years after trial entry a sample of 7000 surviving children (3500 in each group) will be followed up at 2 years of age. This sample will be taken from within the sample recruited during the first 2 years of the project so that follow-up of this group can be completed around the time that the trial stops recruiting. As there seems little possibility that the nature of the effect of the intervention will vary over the duration of trial recruitment, this process will be the most efficient use of resources. For children in the follow-up group, their family will be sent a two part parent-completed questionnaire to assess the child’s health, development and well-being. The first part of the questionnaire comprises the PARCA-R, which has been previously validated as a means of assessing neurodevelopment in a trial setting. The second part focuses on general health issues, and has also been used previously [28, 29]. Major disability at 2 years will be assessed by questionnaires sent to the child’s parents and health care professionals. Major disability will be defined according to the criteria set out in the National Perinatal Epidemiology Unit (NPEU) and Oxford Regional Health Authority document and will include any major disability in the following domains: neuromotor function, seizures, auditory function, communication, visual function, cognitive function and other physical disability [30, 31].

#### Health economics

A prospective economic evaluation will be conducted alongside the trial, with the aim of estimating the cost-effectiveness of the intelligent decision-support software. The economic evaluation will be conducted from a health-service perspective. Information on resource utilisation will be collected through the Guardian® system and hospital-patient administration and maternity information systems. Observational research methods may be used to collect additional costs in intrapartum, postpartum or neonatal care. Current UK unit costs will be applied to each resource item to value total resource use in each arm of the trial. A per diem cost for each level of intrapartum, postnatal and neonatal care will be calculated using NHS reference costs where appropriate, or by sending a detailed questionnaire to the finance department of each centre participating in the trial. The unit costs of clinical events that are unique to this trial will be derived from the hospital accounts of the centres participating in the trial, although primary research may also be required. An incremental cost-effectiveness analysis will be performed and primarily expressed in terms of an incremental cost per poor-perinatal-outcome prevented. The primary outcomes of the trial are likely to have longer-term consequences in terms of health status and health-service utilisation over the mother’s and infant’s lifetime. Consequently, two long term economic evaluations are planned, which in the first instance will estimate the cost-effectiveness of the decision-support software when surviving children reach 2 years of age, and in the second instance will incorporate the lifetime cost and health consequences of the mother and child. To inform the long-term economic evaluations, a subsample of 700 healthy infants selected within the first year of recruitment and all babies with the primary trial outcome who survive to hospital discharge and agree to follow-up will be followed up until 2 years of age or until the end of the trial, whichever comes first. Economic questionnaires completed when the infant reaches 1 and 2 years of age will document the health and social care use of the child and mother. Cost data collected until hospital discharge and from the parental questionnaires will be combined with the PARCA-R composite score at 2 years of age to inform the incremental cost per disability free life years gained when surviving children reach 2 years of age [32]. Given the potential long-term sequelae, these data will then be used to extrapolate long-term outcomes and costs over the child’s lifetime, identifying future health care costs and the health status of mothers and infants from literature as well as the application of decision-analytic or markov methods to synthesise information from different sources. This will require modeling longer-term health-service utilisation from literature and also estimating the potential medico-legal claims that result from adverse events during the intrapartum and neonatal periods.

#### Proposed sample size

The proposed sample size is 46,000 births in total.

The following data sources and assumptions have been used in the calculation of the trial sample size:

#### Incidence of intrapartum stillbirth

This has been estimated as 0.35 per 1000 births. This estimate is derived from the following incidence data: 0.51 per 1000 for all gestations (England, Wales and Northern Ireland, 2004) [33]; 0.27 per 1000 for gestation ≥ 37 weeks (Trent Region, 2002) [34]. This trial is restricting eligibility to women over 35 weeks’ gestation; therefore the incidence will be lower than in women of all gestational ages, which includes those with preterm births. However, it will be higher than for women at term. As women being recruited are all judged to require continuous electronic fetal monitoring it can be assumed that the identification of this “risk group” means that these women are at increased risk of adverse outcomes; therefore the incidence may be higher. In addition these estimates use a denominator of all births which includes women having elective caesarean sections who are not at risk of intrapartum stillbirth as they have no “intrapartum” period. Approximately 7 % of women have elective caesarean section and removal of these women will increase the incidence further. An estimate of 0.35 per 1000 births, therefore, appears reasonable.

#### Incidence of neonatal death

This has been estimated as 0.7 per 1000 births. This estimate is derived from the following data: 3.4 per 1000 for all gestations (England, Wales and Northern Ireland, 2004) [33]; 0.89 per 1000 in those with gestation ≥ 37 weeks (Trent Region, 2005) [32]. This trial is restricting eligibility to women 35 weeks’ gestation or over; therefore the incidence will be lower than amongst women of all gestational ages, which includes those with preterm births. However, it will be higher than for women at term. A reasonable estimate of neonatal death for babies 35 weeks’ gestation or over is therefore 1.0 per 1000 births. Using data from the Trent Survey 2005, 30 % of neonatal deaths were due to congenital anomalies. Therefore this rate can be reduced to 0.7 per 1000 births. As women being recruited are all judged to require continuous electronic fetal monitoring it can be assumed that the identification of this “risk group” means that these women are at increased risk of adverse outcomes; therefore the incidence may be higher. An estimate of 0.7 per 1000 births, therefore, appears reasonable.

#### Incidence of severe and moderate neonatal encephalopathy

The best estimate of the incidence of neonatal encephalopathy in babies born 35 weeks’ gestation or over is 1.3 per 1000 (Trent & Northern Region 2002) [35]. However, as above, women being recruited to this trial are all judged to require continuous electronic fetal monitoring which means that they are at increased risk of adverse outcomes; therefore the incidence may be higher.

#### Combined outcomes

Data are available on some combined outcomes. For example, the incidence of intrapartum stillbirth plus deaths on labour ward assumed to be due to intrapartum asphyxia (the incidence of which is much lower than neonatal mortality) plus severe and moderate neonatal encephalopathy was 1.7 per 1000 (95 % CI: 1.5 to 1.9), range 0.8–2.3 (18 hospitals, Trent 2003–4) and 1.9 per 1000 (95 % CI: 1.6 to 2.3), range 0.6–2.3 (12 hospitals, Yorkshire Neonatal Network 2004–5) [35]. This was for babies born at 35 weeks’ gestation or above with the incidence of these outcomes being higher in the larger hospitals, which attract women with more complicated pregnancies.

#### Incidence of primary outcome for INFANT

We have assumed an incidence of the primary outcome of 3 per 1000 births. This has been calculated by summing the rate of intrapartum stillbirth, neonatal death and moderate and severe encephalopathy which gives an incidence of 2.35 per 1000 births. However, added to this figure is mild encephalopathy, which is reported to occur in 1.25 per 1000 births, and other significant morbidity (other admissions to the neonatal unit within 48 h of birth for ≥ 48 h e.g. feeding difficulties, respiratory symptoms, seizures), for which there are no good estimates of incidence. This estimate of 3 per 1000 births errs on the side of caution and an increased incidence of this outcome in the trial will either (a) increase the power of the trial to demonstrate the same effect size, or (b) allow detection of a smaller effect size with the same trial size, or (c) necessitate a smaller trial if the postulated effect size (or larger) is detected.

### Review of primary outcome

During the early part of the trial, and with advice from the DMC, the primary outcome definition was refined to ensure it captured babies who were likely to have experienced hypoxia during labour. The component of the primary outcome “*Admission to neonatal unit within 48 h of birth for ≥ 48 h with evidence of feeding difficulties, respiratory illness or encephalopathy (where there is evidence of compromise at birth)*” was initially capturing a range of conditions, many of which were unlikely to be related to hypoxia. As a consequence, a process of reviewing each case which fulfilled this component of the primary outcome was implemented. Neonatal unit discharge summaries are collected for all babies admitted to the neonatal unit within 48 h of birth for more than 48 h. For the purposes of data monitoring during the trial, two members of the co-investigator group reviewed all cases which fulfilled this criteria and using a defined data extraction form, ascribed each case as meeting the primary outcome or not. The data extraction form included key elements of the neonatal course which are most likely to be related to intrapartum hypoxia. The form is not an established or validated list of criteria and the numeric scoring was devised to give some quantification of the severity of elements of the clinical course. A score of 3 or greater was agreed to be evidence that the condition of the baby was likely to be associated with intrapartum hypoxia, acknowledging that there remained uncertainty about this, as there is no absolute measure of intrapartum hypoxia. For the final analysis, all cases meeting the broad primary outcome criteria will be reviewed by an independent panel of neonatologists to repeat this process of ascribing each case (masked to allocation) as fulfilling the definition fo the primary outcome or not. Each case will be reviewed (independently) by at least 2 neonatologists and disagreements will be resolved by discussion by the group of five neonatologists.

#### Effect size

The effect size which can be detected with 46,000 women (23,000 in each group), assuming a 5 % level of significance and 90 % power, is a 50 % reduction in poor neonatal outcome rate from 3 to 1.5 per 1000. We have approximated the number of women recruited with the number of infants born, even though women with a twin pregnancy are eligible to join the trial. Approximately 1 in 80 pregnancies are twin pregnancies, however, a proportion of these births will occur before 35 weeks’ gestation and a large proportion of the term births will be by elective caesarean section. We therefore estimate that fewer than 1 % of all births in the study will be twins. In a study of 164 preterm infants [29], the mean (SD) PARCA-R composite score at 2 years was 80 (SD 33) and the mean Mental Development Index (Bayley Scales of Infant Development II) was approximately half a standard deviation below the standardised mean of 100. If we assume that a normal group of term infants would have a PARCA-R composite score half a standard deviation above this sample of preterm infants, then we can estimate a mean (SD) 2 year score of 96 (SD33). Based on this estimate, a follow up sample of size 7000 (3500 per arm) in the INFANT study would have over 90 % power to detect a difference of 3 points in the PARCA-R component score with a two-sided 5 % significance level. The incidence of severe metabolic acidosis (cord-artery pH <7.05) is 10 per 1000 [36–39]. Our proposed sample size will therefore enable us to detect a 28 % relative risk reduction in incidence with over 80 % power in those babies who have their cord artery pH measured.

#### Assumptions

Variations in some of the assumptions of incidence will produce marked variations in the required sample size as the overall incidence is so low. For example, Table 2 illustrates the impact on the required sample size assuming a 5 % level of significance and 90 % power for the same effect size (a 50 % relative risk reduction).

**Table 2:** Sample size assuming 5 % level of significance, 90 % power, 50 % relative risk reduction

Incidence of primary outcome in ‘no decision-support’ group	Incidence of primary outcome in ‘decision-support’ group	Relative risk	Total sample size required
3 per 1000	1.5 per 1000	0.5	46,000
4 per 1000	2 per 1000	0.5	34,000
5 per 1000	2.5 per 1000	0.5	27,000
6 per 1000	3 per 1000	0.5	22,000

#### Primary analysis

The primary analysis will be a comparison of women and babies allocated to “CTG with no decision support” with “CTG with decision support”. Participants will be analysed in the groups into which they were randomly allocated, regardless of allocation received. All women and babies with available data will be included, except for protocol violations and women randomised in error who did not give consent or who were under 16 years of age. The number (percentage) of babies with the composite primary outcome will be presented for each group, and the risk ratio plus 95 % confidence interval (CI) will be calculated. Risk ratios will be estimated using generalised estimating equations (GEE), or a similar method, adjusting for the stratification factors used in the randomisation (centre and singleton/twin pregnancy). This method of analysis will account for the correlation in outcomes between twins and siblings delivered in a subsequent pregnancy during the trial period. A log binomial model will be used in the first instance, but if convergence is not achieved then a log Poisson model will be used with a robust variance estimator [40]. The mean (SD) PARCA-R Composite score will be presented for each group, and the mean difference between groups plus 95 % CI will be calculated and compared using GEE (Gaussian model with identity link). For secondary outcomes including the components of the primary outcome, a 1 % level of statistical significance will be employed. Both adjusted and unadjusted estimates will be presented for all outcomes, but the primary inference will be based on the adjusted analysis.

#### Pre-specified subgroup analysis

To examine whether the effect of decision-support is consistent across specific subgroups of babies, the following subgroup analyses will be undertaken, using the statistical test of interaction:

- Singletons versus twins
- Suspected IUGR at labour onset versus no growth restriction
- BMI group for the subset of women with this recorded: underweight (<18.5); normal (18.5–24.9); overweight (25–29.9); obese (>30); unrecorded
- Centre

#### Economic evaluation

All analyses will be conducted on the basis of intention-to-treat. As the data for costs are likely to be skewed, we shall use non-parametric bootstrap estimation to derive 95 % confidence intervals for mean cost-differences between the trial groups [41]. Non-parametric bootstrap methods will also be used to calculate 95 % confidence intervals for incremental cost-effectiveness ratios. In the absence of stochastic data for all variables, a series of multi-way sensitivity analyses will be undertaken, to explore the implications of uncertainty on the base-case incremental cost-effectiveness ratios. In addition, cost-effectiveness acceptability curves will be constructed using the net-benefits approach [42].

#### Loss to follow-up

Loss to follow-up for the short term primary outcome will be negligible, as most of this information is collected before the woman leaves the delivery room where she has been recruited. For neonatal outcomes of the small number of babies admitted to the neonatal unit, we will collect data for all of these babies through the research midwives employed by the study in the participating centres. For the rare instances where babies are transferred out of the recruiting hospital to another hospital for specialist care, data will be collected from all the hospitals providing care for that child prior to discharge home or death. At the time of entry to the study all women will be asked for permission for their contact details to be downloaded to the trial co-ordinating centre along with their clinical details from the Guardian® system. Families selected for follow-up at 2 years will be contacted by post 8 weeks after birth and informed that they have been selected for the follow-up study. Contact with families who agree to take part will be maintained during the period between birth and the follow-up assessment by sending a birthday card each year along with a FREEPOST change-of-address card to facilitate communication with UCL CTU about updated contact details.

### Trial management

#### Project timetable and milestones

The aim is to randomise 46,000 women to the trial over 36 months. Approximately 320 women per week will therefore need to be recruited. A conservative estimate of the proportion of women who receive EFM in labour and are therefore eligible for trial entry is 60 %.

#### Arrangements for research ethics committee approval

The Investigators will ensure that this study is conducted in accordance with the principles of the current revision of the Declaration of Helsinki (last amended October 2008) and with relevant regulations and with the MRC GCP guidelines which are based on ICH Guidelines for GCP (CPMP/ICH/135/95) July 1996. The trial has National Research Ethics Service – Northern and Yorkshire Research Ethics Committee approval—and local approvals from all the participating centres.

#### Research governance

The sponsor of the trial is UCL. The trial will be run on a day-to-day basis by the UCL CTU Project Management Group. This group reports to the Trial Steering Committee which is responsible to the Research Sponsor (UCL). At each participating centre, local Principal Investigators will report to the Project Management Group via the project funded staff based at the UCL CTU.

#### Insurance

NHS indemnity operates in respect of the clinical treatment being provided. In addition, UCL has appropriate insurance-related arrangements in place in respect of the University’s role as Research Sponsor of this study.

#### Trial steering committee

The trial will be supervised by an independent Trial Steering Committee (TSC). The precise terms of reference for the TSC were agreed at their first meeting. A TSC Charter similar to that used by the DMC (see below) has been completed.

#### Data monitoring committee

An independent Data Monitoring Committee (DMC) has been established for the trial. This is independent of the trial organisers and will meet yearly. The terms of reference for the DMC were agreed at their first meeting. A DMC charter has been completed following the recommendations of the DAMOCLES Study [43].

During the period of recruitment to the trial, interim analyses will be supplied, in strict confidence, to the DMC, together with any other analyses the DMC may request. The data will be supplied to the Chair of the DMC as frequently as they request. Meetings of the committee will be arranged periodically, as considered appropriate by the Chair. In the light of interim data, and other evidence from relevant studies (including updated overviews of the relevant randomised controlled trials), the DMC will inform the TSC, if in their view there is proof beyond reasonable doubt that the data indicate that any part of the protocol under investigation is either clearly indicated or contra-indicated, either for all women or for a particular subgroup of trial participants. A decision to inform the TSC will in part be based on statistical considerations. Appropriate criteria for proof beyond reasonable doubt cannot be specified precisely. A difference of at least 3 standard errors in the interim analysis of a major endpoint may be needed to justify halting, or modifying, such a study prematurely. If this criterion were to be adopted by the DMC, it would have the practical advantage that the exact number of interim analyses would be of little importance, and so no fixed schedule is proposed [44]. Unless modification or cessation of the protocol is recommended by the DMC, the TSC, collaborators and administrative staff (except those who supply the confidential information) will remain blind to the results of the interim analysis. Collaborators and all others associated with the study may write through the trial office to the DMC, to draw attention to any concern they may have about the possibility of harm arising from the treatment under study, or about any other matters that may be relevant.

#### Publication policy

The Chief Investigator will co-ordinate dissemination of data from this study. All publications using data from this study to undertake original analyses will be submitted to the TSC for review before release. To safeguard the scientific integrity of the trial, data from this study will not be presented in public before the main results are published without the prior consent of the TSC. The success of the trial depends on a large number of midwives and obstetricians. For this reason, chief credit for the results will not be given to the committees or central organisers, but to all who have collaborated and participated in the study. Acknowledgement will include all local co-ordinators and collaborators, members of the trial committees, the Trial Co-ordinating Centre and trial staff. Authorship at the head of the primary results paper will take the form “The INFANT Collaborative Group”. This avoids giving undue prominence to any individual. All contributors to the study will be listed at the end of the report, with their contribution to the study identified. Those responsible for other publications reporting specific aspects of the study may wish to utilise a different authorship model, such as “[name], [name] and [name] on behalf of the INFANT Collaborative Group”. Decisions about authorship of additional papers will be discussed and agreed by the trial investigators and the TSC. The women participating in the trial will be sent a summary of the final results of the study, which will contain a reference to the full paper. A copy of the journal article will be available on request.

#### Time period for retention of relevant trial documentation

The policy of UCL CTU is to retain documentation from all its research indefinitely as it is always ‘fit for purpose’. Clinical research in the perinatal period, particularly in relation to new interventions, has the potential to lead to a wide range of unanticipated effects. Thalidomide and diethylstilboestrol are classic examples. Although such effects are most likely with new medicines, we cannot be certain that there will be no long-term consequences of this new technology and that it may become necessary, in the public interest, to undertake long-term follow-up of this cohort of recruited women and children in the future. Any further follow-up will require the necessary approvals before it can go ahead.

## Discussion

This large randomised controlled trial will evaluate whether this intelligent system to support decision making in the management of labour using the cardiotocograph improves perinatal outcome for babies and mothers. By accurately identifying abnormal fetal heart rate patterns associated with perinatal compromise, the system has the potential to refine the use of intrapartum interventions such as emergency caesarean section and instrumental delivery for presumed fetal distress. By correctly identifing the distressed fetus, intervention can be offered in a timely way to improve outcomes, but it may also provide reassurance that the fetus is not compromised and the technology therefore has the potential to decrease unnecessary intervention.

The results of this trial will have importance for pregnant women and for their health professionals.

All changes to this protocol are listed in Table 3.

**Table 3:** Document history

*Version*	*Date*	*Comments*
*1.*	*May 2009*	*Completion of first version.*
*2.*	*Aug 2009*	*Minor edits to text throughout.*
*3.*	*Oct 2009*	*Minor edits to formatting throughout.*
*4.*	*April 2010*	*Minor edits to text.*
*5.*	*May 2011*	*Minor edits to text.*
*6.*	*July 2011*	*Minor edits to text.*
*7.*	*Feb 2012*	*Minor edits to text.*
*8.*	*Mar 2012*	*Minor edits to text.*
*9.*	*May 2012*	*Change of sponsor from University of Oxford to University College London*
*10.*	*Sept 2012*	*Updated contact details for trial staff*
*11.*	*Oct 2012*	*Change wording of primary outcome definition from ‘NICU’ to ‘neonatal unit’.*
*12.*	*Jan 2014*	*Inclusion of primary outcome review process, changes to introduction to match protocol v1, grouping of secondary outcomes as either major diasability or non-major disability*
